# Healthy Volunteers Can Be Phenotyped Using Cutaneous Sensitization Pain Models

**DOI:** 10.1371/journal.pone.0062733

**Published:** 2013-05-09

**Authors:** Mads U. Werner, Karin L. Petersen, Michael C. Rowbotham, Jørgen B. Dahl

**Affiliations:** 1 Multidisciplinary Pain Center, Neuroscience Center, Rigshospitalet, Copenhagen University Hospital, Copenhagen, Denmark; 2 California Pacific Medical Center Research Institute, San Francisco, California, United States of America; 3 Department of Anaesthesia, Centre of Head and Orthopaedics, Copenhagen University Hospital, Rigshospitalet, Copenhagen, Denmark; University of Arizona, United States of America

## Abstract

**Background:**

Human experimental pain models leading to development of secondary hyperalgesia are used to estimate efficacy of analgesics and antihyperalgesics. The ability to develop an area of secondary hyperalgesia varies substantially between subjects, but little is known about the agreement following repeated measurements. The aim of this study was to determine if the areas of secondary hyperalgesia were consistently robust to be useful for phenotyping subjects, based on their pattern of sensitization by the heat pain models.

**Methods:**

We performed post-hoc analyses of 10 completed healthy volunteer studies (n = 342 [409 repeated measurements]). Three different models were used to induce secondary hyperalgesia to monofilament stimulation: the heat/capsaicin sensitization (H/C), the brief thermal sensitization (BTS), and the burn injury (BI) models. Three studies included both the H/C and BTS models.

**Results:**

Within-subject compared to between-subject variability was low, and there was substantial strength of agreement between repeated induction-sessions in most studies. The intraclass correlation coefficient (ICC) improved little with repeated testing beyond two sessions. There was good agreement in categorizing subjects into ‘small area’ (1^st^ quartile [<25%]) and ‘large area’ (4^th^ quartile [>75%]) responders: 56–76% of subjects consistently fell into same ‘small-area’ or ‘large-area’ category on two consecutive study days. There was moderate to substantial agreement between the areas of secondary hyperalgesia induced on the same day using the H/C (forearm) and BTS (thigh) models.

**Conclusion:**

Secondary hyperalgesia induced by experimental heat pain models seem a consistent measure of sensitization in pharmacodynamic and physiological research. The analysis indicates that healthy volunteers can be phenotyped based on their pattern of sensitization by the heat [and heat plus capsaicin] pain models.

## Introduction

It has been hypothesized that the propensity for developing central sensitization in response to noxious events is a heritable trait and may explain differences in susceptibility to developing chronic pain [1]. Noxious skin stimulation of sufficient intensity and duration, leads to the development of an area of secondary hyperalgesia surrounding the stimulation site (cutaneous sensitization). The area of secondary hyperalgesia is experimentally induced with either chemical, electrical or heat stimuli. This phenomenon is inducible in most subjects and thought to be due, at least in part, to central neuronal sensitization [1]. Further, the size of the area of secondary hyperalgesia is commonly used as the primary outcome measure in human experimental studies of pain mechanisms, or when testing the effect of anti-hyperalgesic drugs [2]–[8].

It has been recognized that areas of secondary hyperalgesia varies substantially between subjects, but there is a scarcity of information in the literature about the within-subject and between-subject variability. Human twin studies indicate that genetic factors play an important role in the size of the secondary hyperalgesia area [9]. In support, a recent study of human twins showed that familial effects accounted for 24–32% of observed variances in heat and cold pressor pain thresholds, and, opioid-mediated elevations in cold pressor pain tolerance [10]. These observations need further validation.

To address the variability issue, we pooled drug- and placebo-free data from 10 different healthy volunteer pain model studies in which the area of secondary hyperalgesia, determined with mechanical stimulation, was used as a primary outcome measure. We aimed to examine the robustness of cutaneous secondary hyperalgesia induced by one or more of three different human experimental models: the heat/capsaicin sensitization model (H/C), the brief thermal sensitization model (BTS), and the burn injury model (BI). Because some of the studies used two different models (H/C and BTS) in the same subject, the variability and reliability of the 2 models could be compared directly. Our hypothesis was that it is possible to phenotype subjects based on their pattern of sensitization across days and across models.

## Materials and Methods

Data was obtained from ten studies (9 published [2], [4]–[8], [11]–[13] and 1 unpublished [Petersen et al., unpublished data]) of healthy volunteers (conducted in Copenhagen or San Francisco) in which experimental cutaneous secondary hyperalgesia was induced on two or more study days, and in which the area of secondary hyperalgesia to monofilament stimulation was the primary outcome measure (Table 1). Three different pain models were used: the heat/capsaicin sensitization (H/C) model, the brief thermal sensitization (BTS) model and the burn-injury (BI) model. In the ten studies, five used only the H/C model ([2], [4]–[6], unpublished data), one used the BTS model [11], and one study used the BI model [12]. In three studies subjects underwent both testing with the H/C and BTS model [7], [8], [13]. Study design, timing and procedures were largely similar; differences are noted below and in Table 1. Data collected after administration of a study drug or placebo, were not included in the analyses. In eight studies secondary hyperalgesia areas were measured prior to any study drug or placebo administration, on two or more study days at least one week apart. In two methodology studies not involving any drug administration, secondary hyperalgesia areas were collected during multiple sessions (Table 1) [12], [13].

**Table 1:** Published (2,4–8,11–13) and unpublished volunteer studies on induced secondary hyperalgesia areas.

Author	Ref.	Year	N (female/male)	Age (yrs)	Study aim	Study design	Study model	Number of sessions
**Dirks et al.**	[2]	2000	24 (0/24)	n.a. (21–30)	effect on pain and secondary hyperalgesia by i.v. lidocaine.	DB, R, X, dose-response	Heat/Capsaicin	2
**Petersen et al.**	[4]	2001	14 (5/9)	34 (22–56)	effect on pain and secondary hyperalgesia by i.v. remifentanil.	DB, PC, R, X	Heat/Capsaicin	2
**Dirks et al.**	[5]	2001	25 (0/25)	n.a. (20–30)	effect on pain and secondary hyperalgesia by i.v. adenosine	DB, PC, R, X	Heat/Capsaicin	2
**Mikkelsen et al.**	[6]	2001	25 (0/25)	26 (21–42)	effect on pain and secondary hyperalgesia by i.v. magnesium	DB, PC, R, X	Heat/Capsaicin	2
**Dirks et al.**	[7]	2002	25 (0/25)	n.a. (20–30)	effect on pain and secondary hyperalgesia by gabapentin	DB, PC, R, X	BTS; Heat/Capsaicin	2
**Dirks et al.**	[13]	2003	20 (0/20)	25 (18–44)	methodology: synergy between capsaicin and heat	descriptive	BTS; Heat/Capsaicin	2
**Frymoyer et al.**	[8]	2007	22 (14/8)	27 (22–39)	effect on pain and secondary hyperalgesia by morphine, dextrometorphan	DB, PC, R, X	BTS; Heat/Capsaicin	4
**Petersen et al**.	[11]	2008	53 (23/30)▵	28 (21–51)▵	analgesic tolerance to morphine	DB, PC, P, R	BTS	4
**Ravn et al.**	[12]	2012	100 (50/50)	24 (20–37)	experimental pain sensitivity	descriptive	Burn Injury	2
**Petersen et al.**	-	Unpublished	34 (18/16)	28 (18–54)	effect on pain and secondary hyperalgesia by tramadol, topiramate and gabapentin	PC, CB, DB, X§	Heat/Capsaicin	4

BTS = brief thermal sensitization, CB = complete block design, DB = double-blind, PC = placebo-controlled, P = parallel, R = randomized, X = cross-over.

▵ data from intention-to-treat population,

§ 4-period, 4-treatment, and 4-sequence study.

### Subjects

All subjects were paid healthy volunteers recruited through flyers and advertisements in the Copenhagen and the San Francisco areas. Subjects with use of acute or chronic pain medication, or a prior history of drug or alcohol abuse were excluded. Written informed consent was obtained at the first visit in each study. All studies were conducted in accordance with the Helsinki Declaration and appropriate amendments, and approved by one or more of the following: Ethics Committee of Copenhagen, Danish Data Protection Agency, Danish Medicines Agency (for drug studies), and the Committee on Human Research at the University of California, San Francisco. All subjects were familiarized with the experimental pain model in a pre-study training session.

### Thermal Stimulators and Stimulation Procedures

In six studies heat stimuli were delivered by a MSA thermode (Somedic AB, Hörby, Sweden [size 2.5×5.0 cm^2^]) [2], [4]–[7], [12], [13] and in four studies by a TSA 2001 thermode (Medoc, Ramat Yishai, Israel [size 3.2×4.8 cm^2^])( [4], [11], unpublished data). All procedures were performed with subjects resting head up on a hospital bed, or sitting comfortably in a chair with their arm on the armrest.

### Sensitization Methods

#### Heat/Capsaicin sensitization

The stimulation site was marked with a felt pen on the volar side of the dominant forearm. Sensitization was produced by heating the skin to 45°C for 5 min with the contact thermode. The skin was then covered with capsaicin cream (0.075%) for 30 minutes. After capsaicin removal, the area of secondary hyperalgesia was mapped.

#### Brief thermal sensitization

The stimulation site was marked on the center of the anterior side of the dominant thigh. BTS was induced by heating the skin to 45°C for 3 min. With the thermode still in place (45°C), the borders of secondary hyperalgesia were mapped.

#### Burn injury sensitization

The stimulation site on the medial upper part of the non-dominant, lower leg was marked. A first-degree burn injury, with erythema and hyperalgesia, was induced by applying a contact thermode at 47°C for 7 minutes. The area of secondary hyperalgesia was mapped 1, 2 and 3 hours after induction of the BI.

### Assessment of Secondary Hyperalgesia

The area of secondary hyperalgesia to stimulation with a monofilament (von Frey hair) was the primary outcome measure in all 10 studies. In all but one study the borders of secondary hyperalgesia were determined by stimulating with a monofilament (20.9 g [4], 21.5 g [2], [5]–[7], [13], 26.0 g [8], [11]), along 4 linear paths arranged in 90° angles around the stimulation center, in 5-mm steps at 1-s intervals. Stimulation along each path started well outside the hyperalgesic area and continued toward the stimulated skin area. The subjects, who had their eyes closed during the assessments, reported the occurrence of a definite change in sensation: A mildly noxious pin-like sensation, more intense pricking, burning or tenderness. The border was marked, and the transverse and longitudinal axes were measured for surface area calculations.

In the burn injury study the secondary hyperalgesia border was determined by a monofilament (90.6 g) stimulating in 8 symmetric lines each separated by angles of 45° converging towards the centre of the burn injury. Secondary hyperalgesia areas were measured 1, 2 and 3 hours after the BI. The corners of the octagon were marked on the skin with a felt-tipped pen and transferred to a transparent sheath. The octagonal secondary hyperalgesia area was calculated using a computer-based vector-algorithm (Canvas 12.0, ACD Systems International, Victoria, Canada).

### Statistical Analyses

Parallel analyses were performed for all 3 methods of induction of experimental cutaneous secondary hyperalgesia. Analyses are shown by study, then pooled across all studies. In 4 studies, the data sets no longer contained information on the order of treatment [2], [5]–[7]. For those studies, pre-treatment data from the placebo session was used as the first measurement.

For the continuous outcome of area of secondary hyperalgesia, normality was examined using residual plots and histograms. The strength of association between the first and second measurement was examined with Pearson or Spearman’s correlation coefficients, depending on the distribution. The size of the difference between the 2 measurements was examined with mean differences, tested with a paired t-test. Agreement between the repeat measurements was examined with intraclass correlation coefficients (ICCs (1,1)) and their 95% confidence intervals (CI) were indicated [14]. While there are no agreed upon classifications for levels of agreement for ICCs, for interpretation purposes ICCs were categorized as slight/poor (≤0.2), fair (>0.2 to 0.4), moderate (>0.4 to 0.6), substantial (>0.6 to 0.8) and almost perfect (>0.8) [15]. Bland-Altman plots were presented on pooled data to assess systematic bias in the differences in measurement 1 and measurement 2 [16]. A mixed models approach was used to examine the effect of time in the six studies where the session order was known.

Subjects were categorized in quartiles based on quartile cut-points derived from the distribution of secondary hyperalgesia values from measurement 1 and measurement 2. Some analyses were simplified to three categories, ‘small-’ (1^st^ quartile [<25%]), ‘mid-’ (2^nd^ and 3^rd^ quartile [25–75%]) or ‘large-area’ (4^th^ quartile [>75%]) secondary hyperalgesia). For the repeatability of categorizations, contingency data were evaluated by a quadratic weighted Cohen’s kappa coefficient [17]. For the three studies in which secondary hyperalgesia was induced by both H/C (forearm) and BTS (thigh) on the same day, similar analyses were performed comparing the categorizations from the two methods [7], [8], [13]. Levels of agreement were classified similar to those for ICC listed above.

All significance levels reported are 2-sided. Statistical analyses were conducted using SAS version 9.2 (SAS Institute Inc, Cary, NC) and Medcalc Software version 11.1.6.0 (Mariakerke, Belgium). In the secondary analyses, a Bonferroni correction was applied to *P*-values to examine if the significance observed held after correction for multiple comparisons. The significance level was set to <0.05/9 = 0.006 for the HC analyses, <0.05/5 = 0.01 for the BTS analyses and left as *P*<0.05 for BI analyses because there is only one study with this method of induction. [We do not know of an existing public repository for this type of data. However, data are freely available upon request.].

## Results

**Figure 1:**
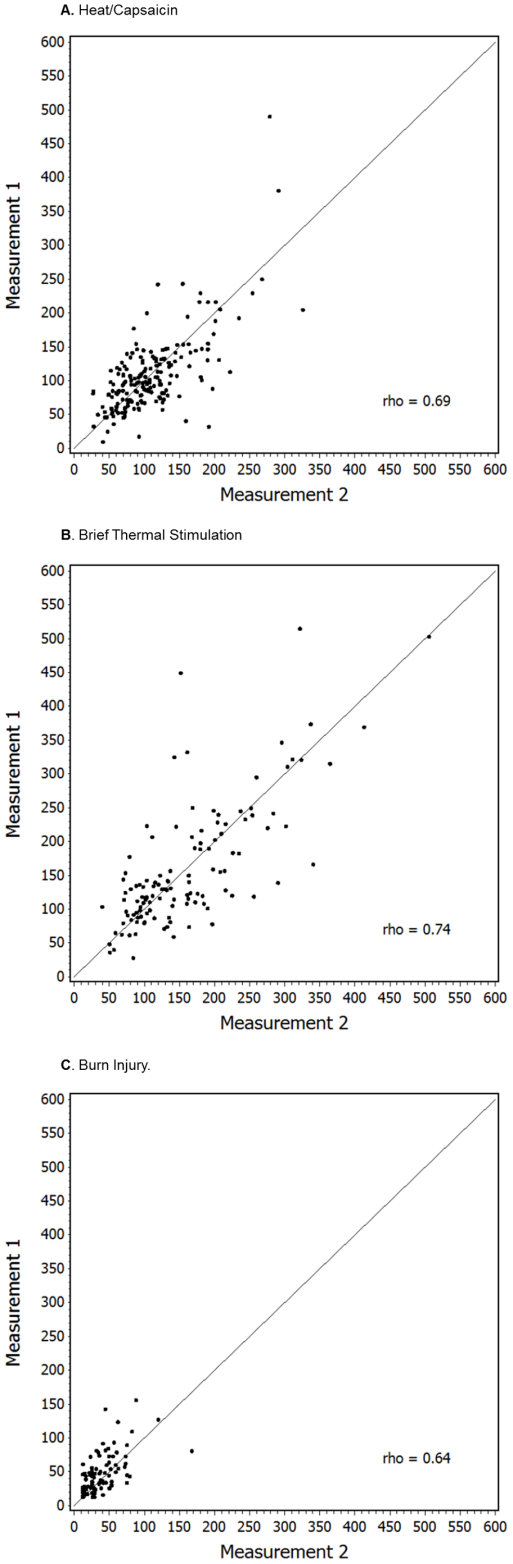
Basic scatterplots of area of secondary hyperalgesia (cm^2^), first vs. second measurement, in the heat/capsacin model (panel A, pooled data from eight studies [n = 189]), the brief thermal stimulation model (panel B, pooled data from four studies [n = 120]) and the burn injury model (panel C, data from one study [n = 100]). The Spearman’s rank correlation coefficient (rho) and line of identity are indicated.

A total of 342 subjects, with 409 repeated measurements, were included in the analyses of the ten studies (Table 1) ([2], [4]–[8], [11]–[13], unpublished data). Five studies used only the heat/capsaicin model (n = 122) ([2], [4]–[6], unpublished data), one study used only the brief thermal sensitization model (n = 53) [11], one study used only the burn injury model (n = 100) [12] and in three studies subjects underwent both the H/C and BTS model (n = 67) [7], [8], [13]. Pooled data on secondary hyperalgesia areas to monofilament stimulation induced with the H/C, BTS and BI models are presented in scatterplots (Figure 1), which show a relatively equal number of points above and below the line of equality between the 2 measurements.

### Reliability of Secondary Hyperalgesia Measurements

Compared to the size of the area of secondary hyperalgesia, the magnitude of the mean difference between measurement 1 and measurement 2 was small and non-significant for all studies, but two (Table 2; BTS [11], BI [12]).

**Table 2:** Areas of secondary hyperalgesia for measurement 1 and 2 (mean +/− SD), *P*-values for paired t-tests between measurements, Spearman’s rank correlation coefficient (rho), coefficient of variation (CV, mean +/− SD), intra-class correlation coefficient (ICC, 95% CI) for 2 measurements and for data with more than 2 measurements.

		Measures of Area of Secondary Hyperalgesia (cm^2^)							2 measurements		>2 measurements
		Measurement 1	Measurement 2		▵Measurement			CV	ICC		ICC
Study	N	mean +/− SD	mean +/− SD		mean +/− SD	*P*	Rho	mean +/− SD	(95% CI)		(95% CI)
***Heat/Capsaicin***											
**Dirks et al.** [2] *	24	99.82+/−41.81	103.20+/−39.49		−3.38+/−40.55	0.69	0.50	20.48+/−12.95	0.52 (0.16, 0.76)		–
**Petersen et al.** [4]	14	141.48+/−76.77	143.28+/−60.64		−1.80+/−49.91	0.89	0.76	16.94+/−14.90	0.76 (0.41, 0.91)		–
**Dirks et al.** [5] *	25	96.04+/−36.81	95.50+/−39.40		0.53+/−29.70	0.93	0.70	19.75+/−18.32	0.71 (0.44, 0.86)		–
**Mikkelsen et al.** [6] *	25	83.16+/−29.85	87.61+/−27.63		−4.45+/−27.50	0.43	0.54	19.30+/−12.68	0.55 (0.21, 0.77)		–
**Dirks et al.** [7] *	25	118.67+/−61.48	134.62+/−46.54		−15.95+/−40.20	0.06	0.76	18.03+/−21.09	0.70 (0.43, 0.86)		–
**Dirks et al.** [13]	20	94.70+/−45.04	97.75+/−43.96		−3.05+/−53.44	0.80	0.28	26.12+/−22.99	0.30 (−0.14, 0.65)		0.66 (0.49, 0.82)
**Frymoyer et al.** [8]	21	86.52+/−41.79	79.02+/−46.77		7.50+/−34.09	0.33	0.71	25.79+/−23.22	0.70 (0.41, 0.87)		0.69 (0.48, 0.84)
**Petersen et al.** §	34	140.75+/−73.54	133.50+/−64.95		7.26+/−57.06	0.46	0.67	19.23+/−15.75	0.67 (0.43, 0.82)		0.68 (0.51, 0.81)
**Pooled**	188	108.08+/−56.75	109.47+/−51.83		−1.38+/−42.84	0.66	0.69	20.60+/−17.91	0.69 (0.61, 0.76)		–
***Brief Thermal Sensitization***											
**Dirks et al.** [7] *	25	198.93+/−108.54	220.64+/−100.88		−21.71+/−55.67	0.06	0.86	14.24+/−18.30	0.84 (0.68, 0.93)		–
**Dirks et al.** [13]	20	201.65+/−125.22	170.20+/−73.41		31.45+/−102.85	0.19	0.57	29.90+/−19.50	0.48 (0.07, 0.76)		0.81 (0.70, 0.90)
**Frymoyer et al.** [8]	22	150.06+/−79.17	128.94+/−83.59		21.12+/−64.01	0.14	0.69	25.87+/−21.34	0.67 (0.37, 0.85)		0.76 (0.59, 0.88)
**Petersen et al.** [11]	53	130.37+/−58.70	142.56+/−63.56		−12.19+/−37.46	0.02	0.82	16.04+/−12.57	0.80 (0.67, 0.88)		0.83 (0.74, 0.89)
**Pooled**	120	160.14+/−92.25	160.94+/−83.75		−0.79+/−63.75	0.89	0.74	19.78+/−17.73	0.74 (0.65, 0.81)		–
***Burn Injury***											
**Ravn et al.** [12]	100	45.03+/−29.39		37.22+/−25.00	7.81+/−24.21	0.002	0.64	28.60+/−21.36		0.58 (0.43, 0.69)	0.71 (0.64, 0.77)

Measure 1 is from Day 1 (or placebo day if no day order was specified) and Measure 2 is the first measurement from the next day where measures were repeated. Measure 1 is the first measurement from Day 1 (or placebo day if no day order was specified) and Measure 2 is the first measurement from the next day where measures were repeated.

§ = unpublished study.

* Measure 1 is from the placebo day, measure 2 is from treatment day. Day order was randomized, so measurement 1 may not have come before measurement 2.

The standard deviations of measurement 1 and measurement 2 for the analysis on pooled data demonstrate the size of the between-subject variation in relation to the average of the measure (Table 2). For H/C the standard deviation was half the size of the average value, for BTS 52 to 57% of the average value, and for BI 65 to 67% of the average value.

### Sensitization Models

#### Heat/capsaicin sensitization model

**Figure 2:**
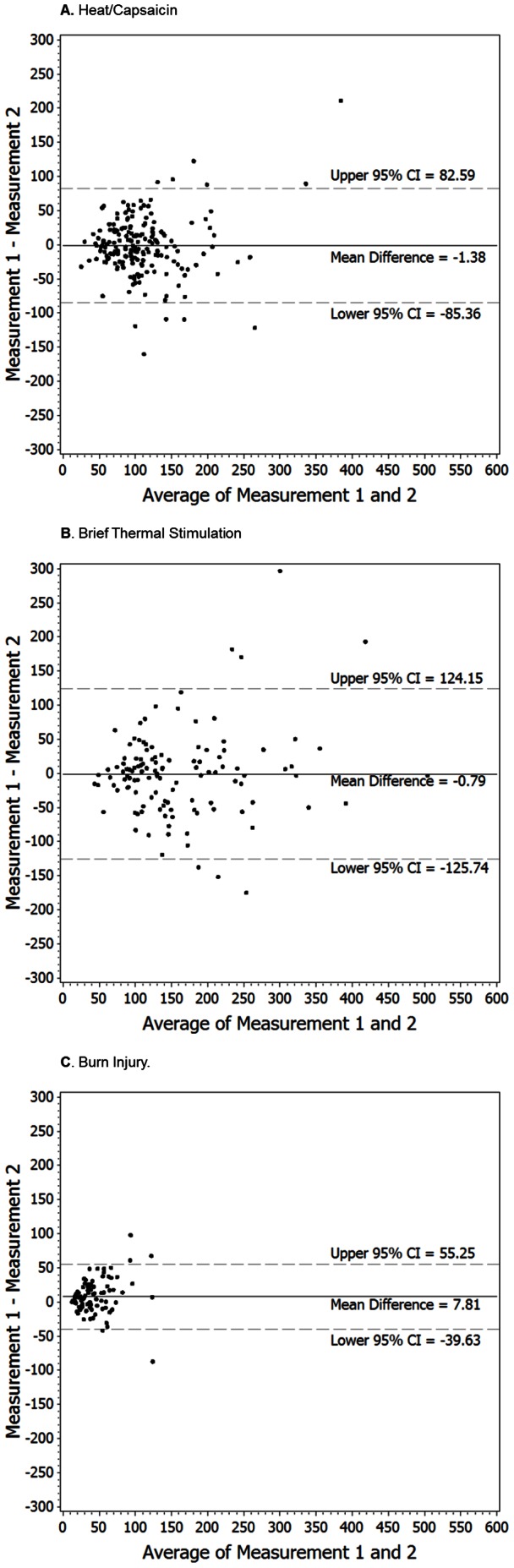
Bland-Altman plots illustrating the mean of measurement 1 and measurement 2 (x-axis) plotted against the difference between measurement 1 and measurement 2 (y-axis) for secondary hyperalgesia areas induced by the heat/capsacin model (panel A, pooled data from eight studies [n = 189]), the brief thermal stimulation model (panel B, pooled data from four studies [n = 120]) and the burn injury model (panel C, data from one study [n = 100]). The solid vertical line (mean of difference) indicate bias between the methods and the dashed vertical lines indicate the upper and lower 95% confidence interval (CI [±1.96×SD = limits of agreement]).

The eight studies ([2], [4]–[8], [13], unpublished data), using the H/C model, had a level of association between the two repeat measurements that was ‘fair’ to ‘substantial’ (rho 0.28 to 0.76), with a correlation coefficient (rho) of 0.69 on the pooled data (Table 2). The mean differences between measurement 1 and 2 ranged from −15.95 cm^2^ to 7.5 cm^2^ and were not statistically, significantly different (*P*≥0.06), showing a small level of disagreement in the pooled analysis (−1.38 cm^2^+/−42.8 cm^2^, *P* = 0.66). The reliability as shown by the ICCs demonstrated ‘fair’ agreement in one study (ICC = 0.30), ‘moderate’ agreement in two studies (0.52 and 0.55), and ‘substantial’ agreement in 5 studies. The analysis on pooled data demonstrated a ‘substantial’ strength of agreement between the 2 measurements (ICC = 0.69). The Bland-Altman plots (Figure 2A) illustrate a random scattering of points both above and below the average mean difference with few points falling outside the 95% confidence interval, showing a lack of systematic bias between the first and second measurements. Mixed models did not show any systematic bias based on whether the measurement was from the first day or a subsequent day (*P* = 0.45).

#### Brief thermal sensitization model

The four studies [7], [8], [11], [13] using the BTS model had a level of association between the 2 repeat measurements that was ‘substantial’ (rho 0.57 to 0.86), with a correlation coefficient (rho) of 0.74 on pooled data (Table 2). The ICCs ranged from 0.48 to 0.84, with one study categorized as ‘moderate’ (0.48) and the remaining 3 studies all categorized as ‘substantial’ agreement or higher (>0.6). The ICC based on pooled data showed ‘substantial’ agreement (0.74). The pooled results demonstrated a small level of disagreement on average (−0.79+/−63.75 cm^2^, N.S.). The Bland-Altman plots (Figure 2B) showed no discernible pattern of systematic bias. Mixed models showed no systematic offset based on day order (*P* = 0.48).

#### Burn injury model

Secondary hyperalgesia induced by the BI model was assessed in one study [12]. The reliability as shown by the ICC was 0.58. There was a significant disagreement on average values (7.81+/−24.21 cm^2^, *P* = 0.002), a bias pattern also evident in the Bland-Altman plot (Figure 2C). The mixed models showed a systematic offset in area of secondary hyperalgesia based on day order, with the second day lower on average (*P* = 0.002). These calculations were based on the one hour post-burn assessment.

Five percent of subjects did not develop an area of secondary hyperalgesia at all (six area measurements collected on two sessions days) and an additional 11% did not develop a secondary hyperalgesia area in ≤4 of the 6 measurements.

#### Categorizations of secondary hyperalgesia areas into quartiles

Agreement between measurements, split up into quartiles, across days and models, is presented in Table 3. Perfect test-retest agreement for all quartiles, in the heat/capsaicin, brief thermal stimulation and the burn injury models, were 51%, 54% and 49%, respectively. Perfect agreement for the 1^st^ quartile (‘small-area’) and 4^th^ quartile (‘large-area’) were between 56 and 76%. The weighted kappa statistics were for the heat/capsaicin model 0.66 (95% CI: 0.57–0.74), the brief thermal stimulation 0.74 (0.65–0.82) and the burn injury model 0.74 (0.65–0.82), corresponding to a good strength of agreement. The probability of measurement 2 moving more than one quartile away from measurement 1 (Table 4) was fairly similar for the three models, ranging from 0 to 13%.

**Table 3:** Agreement of secondary hyperalgesia areas in the heat/capsaicin (**left part of table**), brief thermal sensitization (BTS [**middle part of table**]) and the burn injury (**right part of model**) model, at **Measurement** **1** (rows) and **Measurement 2** (columns).

		Heat/capsaicin					Brief Thermal Sensitization					Burn injury				
		Measurement 2					Measurement 2					Measurement 2				
		<25%	25–50%	51–75%	>75%	Sum	<25%	25–50%	51–75%	>75%	Sum	<25%	25–50%	51–75%	>75%	Sum
**Measurement 1**	**<25%**	31	12	4	1	**48**	21	7	2	0	**30**	16	9	0	0	**25**
	**25–50%**	11	16	16	5	**48**	7	10	11	2	**30**	7	10	5	3	**25**
	**51–75%**	5	14	18	9	**46**	2	11	12	5	**30**	2	6	9	8	**25**
	**>75%**	0	6	10	31	**47**	0	2	6	22	**30**	0	0	11	14	**25**
		**47**	**48**	**48**	**46**	**189**	**30**	**30**	**31**	**29**	**120**	**25**	**25**	**25**	**25**	**100**

Data indicate the distribution of observations split into quartiles (<25%, 25–50%, 51–75%, >75%) giving a 4×4 contingency table (total number of observations for each quartile are indicated in lower row [Measurement 2] and right-hand column [Measurement 1]). The numbers in the lower right-hand corner, in each panel, are the total number of observations. The numbers in bold indicate completely agreement between observations: Measurement 1 and Measurement 2. Perfect agreement for the heat/capsaicin (**A**), brief thermal stimulation (**B**) and the burn injury (**C**) models were seen in 51%, 54% and 49% of the observations, respectively. The weighted Cohen’s kappa statistics were 0.66 (95% CI: 0.57–0.74) for the heat/capsaicin model, 0.74 (0.65–0.82) for the brief thermal stimulation and 0.74 (0.65–0.82) for the burn injury model.

**Table 4:** The table illustrates the probability of measurement 2 moving more than one quartile away from measurement 1 and vice versa for the heat/capsaicin, brief thermal sensitization (BTS) and burn injury models.

		Heat/capsaicin		BTS		Burn injury	
		≥50%	<50%	≥50%	<50%	≥50%	<50%
**<25%**	**Measurement 1**	5/48	**43/48**	2/30	**28/30**	0/25	**25/25**
	**Measurement 2**	5/47	**42/47**	2/30	**28/30**	2/25	**23/25**
**>75%**	**Measurement 1**	**41/47**	6/47	**28/30**	2/30	**25/25**	0/25
	**Measurement 2**	**40/46**	6/46	**27/29**	2/29	**22/25**	3/25

The distribution of the lowest (<25%) and highest (>75%) quartiles (rows [split into **Measurement 1** and **Measurement 2** observations]) divided in the 2 higher (≥50%) and the 2 lower (<50%) quartiles (columns), across the 3 different models. As an example: in the heat/capsaicin model the number of observations **Measurement 1** with areas in the highest quartile (>75% [n = 47]) corresponded at **Measurement 2** to 41 observations at or above the median (≥50%) and 6 observations below median value (<50%).

#### Repeatability across models (H/C and BTS)

In order to simplify calculations the observations were categorized in ‘small-area’ (1^st^ quartile), ‘mid-area’ (2^nd^ and 3^rd^ quartile) and ‘large-area’ (4^th^ quartile) secondary hyperalgesia (Table 4). There were three studies [5], [7], [8] with two measurements of secondary hyperalgesia, induced by both HC and BTS that were measured concurrently. The agreement in classification to ‘small-’, ‘mid-’ or ‘large-areas’ across induction method, was 59% for measurement 1 and 69% for measurement 2 (Table 5). When assessed with both the H/C and BTS on the first assessment day, 32% of the subjects fell in either ‘small-area’ or ‘large-area’ categories with both models. Similarly 42% fell in the same category on the second assessment day (Table 5). The weighted kappa statistics for these six measures ranged from 0.24 to 0.67, with 5 (62.5%) of the comparisons having ‘moderate’ or ‘substantial’ agreement (Table 6).

**Table 5:** Comparing the area categorizations (small, mid, large) between the heat/capsaicin and the brief thermal sensitization methods when measured on the same subject at the same time.

Pooled						
	*Brief Thermal Sensitization*					
	Measurement 1			Measurement 2		
*Heat/Capsaicin*	Small-area	Mid-area	Large-area	Small-area	Mid-area	Large-area
**Small-area**	8 (12.1%)	8 (12.1%)	3 (4.6%)	17 (25.4%)	7 (10.4%)	0
**Mid-area**	5 (7.6%)	18 (27.3%)	9 (13.6%)	0	18 (26.9%)	10(14.9%)
**Large-area**	0	2 (3.0%)	13 (19.7%)	0	4 (6.0%)	11 (16.4%)
Perfect agreement:	39 (59.0%)			46 (68.7%)		
Agreement moving >1 level:	3 (4.6%)			0 (0%)		

The agreement in classification to small-, mid- or large areas across induction methods was 59% for measurement 1 and 69% for measurement 2.

**Table 6:** The weighted Cohen’s kappa statistic for the pooled analyses showed moderate agreement for measurement 1 (0.44) and substantial agreement for measurement 2 (0.62).

		Measurement 1		Measurement 2	
		Weighted		Weighted	
**Study**	**N**	**Kappa (95% CI)**	***P*** **-value**	**Kappa (95% CI)**	***P*** **-value**
**Dirks et al.** [7]	25	0.48(0.23,0.74)	0.01	0.67 (0.41,0.93)	<0.0001
**Dirks et al.** [13]	20	0.49 (0.21,0.77)	0.002	0.34 (0.03,0.65)	0.03
**Frymoyer et al.** [8]	22	0.24 (−0.09,0.58)	0.1	0.57 (0.32,0.84)	0.0003
**Pooled**	**67**	**0.44 (0.27,0.61)**	**<0.0001**	**0.62 (0.48,0.76)**	**<0.0001**

The kappa statistics were significantly different from zero (what would be expected from chance alone) for 1 comparison.

#### Value of additional measurements

Including additional measurements in the ICC calculations for the three studies [5], [7], [8] with >2 days of data, had little effect, except for one study with a low ICC comparing the measurements on the first 2 days, which yielded an improvement in ICC when an additional 4 measurements were added (H/C: 0.30 vs. 0.66; BTS: 0.48 vs 0.81) [5]. In the BI study, using the median values of the 3 hourly post-burn assessments, increases in the correlation coefficient (rho) from 0.64 to 0.77 (95% CI, 0.68–0.84) and for the ICC from 0.58 to 0.67 (95% CI, 0.52 to 0.77), were seen.

#### Secondary statistical analyses

Results were largely similar after applying a Bonferroni correction to *P*-values. The mean difference in area of secondary hyperalgesia for the BTS method was no longer significantly different from zero, and most of the significance testing that the kappa statistics were different from zero lost significance.

## Discussion

This study demonstrated that measurement of secondary hyperalgesia areas, induced by experimental heat [and heat plus capsaicin] pain models, seems a consistent and repeatable measure of sensitization in pharmacodynamic and physiological research. The statistical analyses indicated that healthy volunteers can be phenotyped based on their pattern of sensitization by the heat pain models, confirming the study hypothesis.

### Within-Subject vs. Between-Subject Variation

Most importantly, the present analysis showed a low within-subject test-retest variation, compared to the substantial between-subject variation. Intraclass correlation coefficients indicated that the within-subject variance was approximately 25% of the between-subject variance demonstrating a good within-subject agreement during repeated assessments.

### Agreement across Measurements and Methods

The agreement between measurements, in pooled secondary hyperalgesia areas, was ‘substantial’ in all models and the agreement between areas induced with the H/C and BTS models on the same study day was ‘moderate’ to ‘substantial’(Table 2). Grouping the first measurement session data into quartiles, widely separating the ‘small-area’ from ‘large-area’ responders, illustrated that the categorizations generally held up at the second measurement session (Table 3 and 4). For ‘small-area’ (1^st^ quartile) as well as ‘large-area’ (4^th^ quartile) responders, 56–76% of subjects remained in the same quartile grouping. Subjects almost never crossed all the way from ‘small-area’ to ‘large-area’ (or vice versa), this was only seen in 1 out of 409 assessments. Surprisingly, for those studies that had more than two sessions, the improvement of ICC from having additional measurement sessions was limited.

These results suggest that the area of secondary hyperalgesia is reproducible across multiple sessions. ‘Small-area’ and ‘large-area’ responders can be selected with reasonable confidence by requiring that a subject fall in the respective categories on two consecutive model inductions. If the goal in a pharmacodynamic trial was to select a group of 20 ‘small-area’ and 20 ‘large-area’ responders, approximately 130 subjects would be needed to undergo pre-trial induction of secondary hyperalgesia on the first measurement. From these tested subjects ‘small-area’ and ‘large-area’ responders are selected (n = 65) and continue with the same model on the second measurement. Since 60–70% will repeat an identical outcome at second measurement, at least 20 ‘small-area’ and 20 ‘large-area’ subjects with a consistent secondary hyperalgesia response are likely to appear. Noteworthy, when secondary hyperalgesia was induced with both the H/C (forearm) and BTS (thigh) in the same subject on the first assessment day, 32% of subjects were classified in the same responder group (‘small' or ‘large') with both models, suggesting robustness across models, and that subjects can be classified as ‘small’ or ‘large’ area subjects in a single study day.

### ‘No-Responders'

Little information is available on the proportion of healthy persons in the general population who would fail to develop an area of secondary hyperalgesia in response to one of the three pain models used in the 10 studies analyzed here. For the 9 studies using H/C and/or BTS ([2], [4]–[8], [11], [13], unpublished data), such subjects were excluded after the training session for failing to develop the primary outcome measure and no further data were collected. In the BI study [12] analyzed, 5% failed to develop an area at any session.

### Limitations and Advantages of the Study

*First*, this is a secondary analysis of 9 published and one unpublished study, which should be taken into consideration, when interpreting the results. The study is, as such, a hypothesis generating study, but the high number of subjects (n = 342) and test-retest measurements (409 repeated measurements) seem to make the hypothesis solidly based. *Second*, the studies differ in methodology in regard to thermode size, measurement timing, bending force of the monofilaments, area calculations and body region sensitized. Some of these variables may affect the outcome, but unfortunately there is a void in the literature on the significance of these interactions. In each study, the methods were identical during the test-retest measurements, counterbalancing some of the negative effects by pooling the studies. An illustrative example was seen in the BI-model where a highly significant carry-over effect across the measurements was evident, indicating a habituation effect between measurements (Figure 2C). The median interval between the measurements was 3 weeks, but hyposensitivity to painful stimuli has been described for up to 6 weeks after the BI [18]. Carry-over effects were not observed with either the H/C or the BTS pain models. To counterbalance this effect in the BI-model we refrained from using absolute area values, but used a relative ranking of the values during each of the two measurements, which seemed to eliminate the effect of habituation. Further, the robustness of the results across models and trial methodology may be considered to strengthen the overall conclusion of the analyses. *Third*, secondary hyperalgesia may differ not only in area-size but also in regard to duration [19], but the present study, based on single time measurements is not accommodated to address this question. *Fourth*, it is important to realize that data from the present study in volunteers, cannot be extrapolated to clinical situations with patients.

### Phenotyping and Prediction of Pain Sensitivity

Although speculative, our findings may represent a window of opportunity to further explore differences (phenotypes) in individual pain sensitivity and propensity to develop areas of secondary hyperalgesia, which likely depends on central neuronal sensitization. As pointed out by Woolf [1], the comorbidity of a number of pain hypersensitivity syndromes and their similar pattern of clinical presentation may reflect inherent, common attributes of central sensitization to their pathophysiology. Most importantly, the potential predictive value of identifying ‘small-’ and ‘large-area’ responders needs to be explored. Thus far, one study comprising 20 patients investigated the potential of preoperatively induced secondary hyperalgesia to predict subsequent dynamic postoperative pain in surgical patients, but no correlations were found [20]. However, a recent study demonstrated postsurgical area of secondary hyperalgesia to predict chronic postsurgical neuropathic pain in the iliac crest bone harvest model [21]. Determining the predictive value of secondary hyperalgesia area for acute pain and other measures of pain hypersensitivity, as well as the risk for development of chronic pain, await much larger sufficiently powered studies.

### Phenotyping and Cutaneous Sensitization

The results of the previously mentioned studies in twins [9], [10] demonstrate substantial influence of heritable factors for response to pain stimuli, such as the cold pressor response. A subject’s propensity subject of developing cutaneous sensitization may also contain underlying genetic factors that can be explored [9]. Phenotyping subjects based on their the ability to develop cutaneous sensitization may, in fact, be equally or more important than phenotyping subjects on the basis of pain thresholds and could potentially be added to the battery of testing following algorithms like those of the German Research Network on Neuropathic Pain (DFNS) [22].

### Conclusion

In conclusion, post-hoc analyses of test-retest data from 342 healthy volunteers in 10 studies, employing experimentally induced cutaneous secondary hyperalgesia, in addition to corroborating the methodological consistency of these models in physiological and pharmacodynamic research, indicated that healthy volunteers can be phenotyped based on their pattern of sensitization by the heat [and heat plus capsaicin] pain models. These findings may represent an incentive to further explore individual differences in ability to develop neuronal sensitization, and to investigate if ‘large-area' responders are at an increased risk of developing conditions associated with pain hypersensitivity and chronification – and whether the pharmacodynamic profiles of analgesics and anti-hyperalgesics differ between ‘large-area' and ‘small-area' responders.
